# Predicting Therapy Outcomes in Patients With Stress-Related Disorders: Protocol for a Predictive Modeling Study

**DOI:** 10.2196/65790

**Published:** 2025-03-25

**Authors:** Ludwig Franke Föyen, Victoria Sennerstam, Evelina Kontio, Oskar Flygare, Magnus Boman, Elin Lindsäter

**Affiliations:** 1 Division of Psychology Department of Clinical Neuroscience Karolinska Institutet Stockholm Sweden; 2 Stress Research Institute Department of Psychology Stockholm University Stockholm Sweden; 3 Gustavsberg University Primary Care Center Academic Primary Care Center Region Stockholm Stockholm Sweden; 4 Department of Clinical Neuroscience Osher Center for Integrative Health Karolinska Institutet Stockholm Sweden; 5 Centre for Psychiatry Research Department of Clinical Neuroscience Karolinska Institutet and Stockholm Health Care Services Stockholm Sweden; 6 Division of Clinical Epidemiology Department of Medicine Solna Karolinska Institutet Stockholm Sweden; 7 BioClinicum MedTechLabs Karolinska University Hospital Stockholm Sweden; 8 Division of Psychiatry University College London London United Kingdom

**Keywords:** adjustment disorder, cognitive behavioral therapy, exhaustion disorder, machine learning, predictive modeling, psychological stress, therapy outcome

## Abstract

**Background:**

While cognitive behavioral therapy has shown efficacy in treating stress-related disorders, such as adjustment disorder and exhaustion disorder, knowledge about factors contributing to treatment response is limited. Improved identification of such factors could enhance assessment procedures and treatment strategies. In addition, evaluating how traditional prediction methods and machine learning can complement each other may help bridge gaps in understanding and predicting treatment response.

**Objective:**

This study aims to (1) evaluate putative predictors of treatment response in patients with stress-related disorders using traditional prediction methods and (2) model treatment outcomes using a machine learning approach. This design combines the interpretability of traditional methods with the ability of machine learning to identify complex patterns.

**Methods:**

We will analyze data from a randomized controlled trial comparing 2 internet-delivered treatments, cognitive behavioral therapy versus an active control treatment, for patients diagnosed with adjustment disorder or exhaustion disorder (N=300). Prediction models will be based on pooled data from both treatment arms due to the limited sample size and lack of knowledge on predictors of treatment effects. Putative predictors include sociodemographic and clinical information, clinician-assessed data, self-rated symptoms, and cognitive test scores. The primary outcome of interest is responder status on the Perceived Stress Scale-10, evaluated based on the reliable change index posttreatment. For the traditional approach, univariate logistic regressions will be conducted for each predictor, followed by an ablation study for significant predictors. For the machine learning approach, 4 classifiers (logistic regression with elastic net, random forest, support vector machine, and AdaBoost) will be trained and evaluated. The dataset will be split into training (70%) and testing (30%) sets. Hyperparameter tuning will be conducted using 5-fold cross-validation with randomized search. Model performance will be assessed using balanced accuracy, precision, recall, and area under the curve.

**Results:**

All data were collected between April 2021 and September 2022. We hypothesize that key predictors will include younger age, education level, baseline symptom severity, treatment credibility, and history of sickness absence. We anticipate that the machine learning models will outperform a dummy model predicting the majority class and achieve a balanced accuracy of ≥67%, thus indicating clinical usefulness.

**Conclusions:**

This study will contribute to the limited research on predictors of treatment outcome in stress-related disorders. The findings could support the development of more personalized and effective treatments for individuals diagnosed with adjustment disorder or exhaustion disorder, potentially improving clinical practice and patient outcomes. If successful, this dual approach may encourage future studies with larger datasets and the implementation of machine learning models in clinical settings, ultimately enhancing precision in mental health care.

**International Registered Report Identifier (IRRID):**

DERR1-10.2196/65790

## Introduction

### Background

Mental disorders have a negative effect on quality of life, often precipitating personal suffering and work disability [1]. Around 23% of all who receive a psychiatric diagnosis in Swedish primary care receive a stress-related diagnosis [2], and these account for most psychiatric long-term sickness absences [3]. In Sweden, disorders believed to stem from persistent or overwhelming subtraumatic life events are often categorized using the diagnostic labels adjustment disorder (AD) or exhaustion disorder (ED). Even though ED is only recognized as a medical diagnosis in the Swedish version of the *International Classification of Diseases, Tenth Revision*, the clinical picture of ED is similar to the internationally acknowledged burnout construct [4], a condition that is often associated with significant suffering and work disability [5].

According to diagnostic definitions of AD and ED, these conditions develop in the context of one or several subtraumatic life events (stressors), resulting in mixed symptoms of anxiety, depressed mood, disturbed sleep, fatigue, and impaired memory and concentration. They share symptomatology with other mental disorders, and their diagnostic validity is debated [6,7]. Despite evidence indicating the efficacy of cognitive behavioral therapy (CBT) [8-10] and problem-solving interventions [11] on symptoms of stress, many studies have suffered from significant attrition, and knowledge regarding the factors that contribute to treatment response is still limited [8,12]. Improved identification of such factors could facilitate development of improved assessment procedures and adaptive treatment strategies that might improve outcomes [13].

Research on predictors of psychiatric treatment outcomes is limited [14,15] but demographic factors (eg, age and education level) [16,17], clinical characteristics (eg, use of medication and symptom severity) [17-19], treatment-related factors (eg, treatment credibility and adherence) [16,18] and cognitive functioning [20] have been associated with treatment outcomes.

When it comes to studies investigating predictors of treatment for stress-related disorders, Kocalevent et al [15] found that symptoms of anxiety but not perceived stress, depressive symptoms, or demographic variables predicted self-rated mental health following treatment for patients diagnosed with AD. In a study investigating burnout, Pallich et al [21] identified emotional competence, but not demographic characteristics, as a predictor of treatment response. However, both of these studies suffer from limited generalizability due to their inadequate description of the treatment offered, the fact that the interventions were conducted in an inpatient setting, and the lack of control groups. In ED patients, one study identified several predictors of treatment outcome following multimodal rehabilitation, including younger age, baseline symptom severity (insomnia, anxiety, and depression), perfectionism, physical activity level, treatment credibility, and a history of sickness absence due to ED [22]. However, the effects of demographics and pretreatment symptoms were so small that they offered limited clinical utility. In sum, at the current stage of research, it is a challenge for clinicians to determine who will benefit from treatment, underscoring the imperative for more sophisticated predictive studies.

Traditionally, prediction in psychiatry has relied on interpretable linear or logistic regression models. The aim has been to identify variables explaining a statistically significant portion of the variance in outcome, under the premise that such variables should inform researchers and clinicians. For example, the presence of previous sickness absence and earlier unsuccessful treatment attempts might lead a psychologist to conclude that a patient requires additional support, possibly extending the treatment duration. Although this approach of identifying predictors has offered some clinical utility, it often falls short in practice; the predictive power of specific variables in isolation is typically inadequate to inform assessment, treatment selection, and adaptations of interventions. Given the inherent complexity of mental disorders, the likelihood of pinpointing strong predictors with clinical utility is small, thus limiting the practical value of this approach [23,24].

Machine learning (ML) represents a promising methodological shift in psychiatric prediction modeling, transitioning from the identification of statistically significant predictors to an emphasis on quantifiable model performance, characterized by ensemble methods and adaptability to new datasets. This approach often sacrifices explainability in favor of enhanced predictive performance but offers unique value in handling the complex, nonlinear, high-dimensional data characteristic of mental disorders [25]. With this approach, a model generates a prediction (eg, remission, yes or no) intended to be actionable for a clinician. For example, patients predicted to have low probability of treatment success could be offered additional psychological support or an alternative intervention, thus increasing the likelihood of remission [26,27].

Forsell et al [28] have proposed a balanced accuracy (BACC) threshold of 67% as a benchmark for clinical utility in psychiatric applications, offering a tangible goal for ML implementation. However, the efficacy of ML in this domain remains an ongoing area of inquiry, and its capacity to surpass conventional methods in clinical usefulness is yet to be established [29].

Given the high prevalence and substantial societal costs associated with stress-related disorders, it is imperative to critically evaluate both the applicability and the limitations of ML within this specific context. Such an assessment will not only contribute to the broader understanding of the role of ML in precision psychiatry but also inform the development of more effective diagnostic and treatment strategies for stress-related disorders.

### Objective of the Study

The overall objective of this study is to predict treatment outcomes in patients with stress-related disorders. Due to limitations in existing methods for prediction analyses, this study aims to first evaluate putative predictors using a traditional prediction paradigm, and second to model treatment outcomes using an ML approach. Our primary outcome of interest is responder status after treatment on the Perceived Stress Scale-10 (PSS-10), evaluated using the reliable change index (RCI; further described in the Planned Statistical Analysis and Data Cleaning and Preparation sections). On the basis of earlier research on predictors of treatment outcome, we hypothesize that key predictors will include younger age, education level, baseline symptom severity, treatment credibility, and history of sickness absence. Furthermore, we anticipate that the ML models will outperform a dummy model predicting the majority class and achieve a BACC of ≥67%, thus being indicated clinically useful [28].

## Methods

### Study Design

We will use collected data from a randomized controlled trial (RCT; N=300) of internet-delivered CBT for patients diagnosed with AD or ED compared to an active, internet-delivered control condition consisting of general health-promoting advice. A priori power analysis conducted for the main outcome in the RCT indicated that 300 study participants would be needed for a 90% power to detect a between-group effect size of Cohen *d*=0.4 with a significance level of .05 and an expected attrition rate of 10%. Due to the limited sample size and general lack of knowledge on predictors of treatment effect, prediction models in this study will be based on pooled data from both treatment arms. The study design is prospective, and predictors will include sociodemographic and clinical information, clinician-assessed data, self-rated symptoms, and results from cognitive test scores. The results will be reported in line with the TRIPOD+AI (Transparent Reporting of a Multivariable Prediction Model for Individual Prognosis or Diagnosis+Artificial Intelligence) statement [30].

### Ethical Considerations

The study was approved by the Swedish Ethical Review Authority (registration 2020–03198; 2023–06857-02) and was preregistered on ClinicalTrials.gov (NCT04797273). All participants provided written informed consent before inclusion, and their data are pseudonymized and securely stored on an encrypted server. Participants received no monetary compensation but accessed study interventions free of charge. No identifying information of participants will be included in the manuscript or supplementary materials.

### Procedure

#### Participants

In total, 300 nationally recruited individuals were diagnosed with a primary diagnosis of AD (n=142, 47.3%) or ED (n=158, 52.7%) and were included in the RCT. Participant recruitment was carried out through social media, newspaper advertisements, and information provided to health care clinics. Participants self-referred to the study web page, where they signed digital informed consent and completed a screening battery consisting of sociodemographic and clinical background questions as well as self-report symptom questionnaires. Participants were subsequently clinically assessed by a psychologist using a structured diagnostic interview, including Mini International Neuropsychiatric Interview [31], self-rated ED [32], and the Adjustment Disorder New Module-8 (ADNM-8) [33]. For inclusion, participants needed to (1) fulfill the criteria for a primary diagnosis of AD or ED, (2) be aged between 18 and 65 years, (3) have regular access to a computer with internet access, and (4) be able to read and write in the Swedish language. Exclusion criteria included (1) drug use or addiction during the past 6 months, (2) current or past psychosis or bipolar disorder, (3) current risk of suicide, (4) changed psychopharmacological treatment in the past month, (5) other ongoing psychological treatment, and (6) previous experience of CBT for AD or ED in the past year.

#### Treatment

Patients were randomized to one out of two 12-week internet-delivered treatments (CBT and general health-promoting advice). They both consisted of web-based text-based modules with related exercises and assignments. Patients were guided sequentially through the modules by a therapist via a secure web-based platform. The therapists’ primary role was to provide feedback on exercises, support in problem-solving, and to give emotional and technical support via weekly asynchronous text messages. Therapists were licensed clinical psychologists or clinical psychology students in their final year of training. Because this study will not evaluate the individual treatments, they will not be further described here. A full description of the treatments is described in the study by Sennerstam et al [34].

### Outcomes

The primary outcome in this study and the original RCT is PSS-10 [35]. The PSS-10 is a self-report questionnaire developed to evaluate an individual’s perception of life as unpredictable, uncontrollable, and overwhelming. Responses are recorded on an ordinal scale ranging from 0 n*ever* to 4 *very often*, reflecting the individual’s feelings and thoughts over the past month. It contains statements, such as ‘*In the last month, how often have you been upset because of something that happened unexpectedly?’* and sum scores range from 0 to 40. The PSS is the most commonly used outcome measure of stress-management interventions globally [8,36,37]. For this study, a Swedish version of the PSS-10 was used. The PSS-10 has been found to exhibit high internal consistency (Cronbach α=0.84) and adequate construct validity [38]. The PSS-10 was administered digitally through the web-based study platform before randomization to treatment, every 3 weeks during the treatment phase, and at treatment completion (12 weeks). During treatment, the instructions for the PSS-10 were modified to have patients consider the last week instead of the last month. For this study, the sum score of the PSS-10 will be dichotomized into responder or nonresponder after treatment based on the RCI criteria [39] to differentiate between statistically significant change and those attributable to measurement error or natural variability. The PSS-10 baseline and 3-week measurement will also be used as predictors.

### Putative Predictors

#### Overview

Predictors were gathered through self-report measures that were administered in the web-based study platform, clinical assessment conducted before inclusion to the study, and remote cognitive testing. Table 1 presents all predictors included in the study.

**Table 1:** Putative predictors of treatment outcome in stress-related disorders

Predictor		Construct measured	Type	Clinician-rated	Scoring range
**Sociodemographics**					
	Age (y)	—^a^	Interval		18-65
	Sex	—	Categorical		Male or female
	Relationship status	—	Categorical		3 categories
	Number of children	—	Interval		0-∞
	Educational attainment	—	Ordinal		9 categories
	Employment status	—	Categorical		8 categories
	Employment type	—	Categorical		11 categories
	Self-rated computer skills	—	Ordinal		5 categories
	Self-rated reading skills	—	Ordinal		5 categories
	Swedish native speaker	—	Categorical		Yes or no
**Clinical characteristics**					
	Number of medications	Medication	Interval	✓	0-4
	Antidepressants	Medication	Categorical	✓	Yes or no
	Sleep medication	Medication	Categorical	✓	Yes or no
	Pain medication	Medication	Categorical	✓	Yes or no
	Anxiolytics	Medication	Categorical	✓	Yes or no
	Diagnosis	Primary diagnosis	Categorical	✓	2 categories
	Secondary diagnosis	Secondary diagnosis	Interval	✓	0-4
	Depression	Secondary diagnosis	Categorical	✓	Yes or no
	Anxiety disorder	Secondary diagnosis	Categorical	✓	Yes or no
	Insomnia	Secondary diagnosis	Categorical	✓	Yes or no
	Other disorders	Secondary diagnosis	Categorical	✓	Yes or no
	S-ED^b^	Exhaustion disorder	Ordinal	✓	3 categories
	ADNM-8^c^ criteria	Adjustment disorder	Categorical	✓	Yes or no
	ADNM-8 number of stressors	Adjustment disorder	Interval	✓	0-11
	ADNM-8 stressors	Adjustment disorder	Categorical	✓	16 categories
	Duration of current episode	—	Interval	✓	0-∞
	Age of first episode (y)	—	Interval	✓	0-65
	Sick-leave status	Sickness absence	Interval		0%-100% 5 steps
	Sick-leave duration	Sickness absence	Ordinal		5 categories
**Self-rated symptoms**					
	AUDIT^d^	Alcohol consumption	Interval		0-40
	GAD-7^e^	Anxiety symptoms	Interval		0-21
	SMBQ^f^ cognitive weariness	Burnout	Continuous		0-7
	SMBQ exhaustion	Burnout	Continuous		0-7
	SMBQ listlessness	Burnout	Continuous		0-7
	MADRS-S^g^	Depression	Interval		0-54
	KEDS^h^	Exhaustion disorder	Interval		0-54
	WHODAS^i^ 2.0	Functional disability	Continuous		0%-100%
	EQ-5D-5L	Quality of Life	Interval		5-25
	BBQ^j^	Quality of life	Interval		0-96
	ISI^k^	Insomnia severity	Interval		0-28
	SRH-5^l^	Self-rated health	Interval		0-5
	PSS-10^m^	Perceived stress	Interval		0-40
	PHQ-15^n^	Somatoform symptoms	Interval		0-30
	6-QEMP^o^	Subjective memory impairment	Interval		0-30
**3-week measurement**					
	SMBQ cognitive weariness	Burnout	Continuous		0-7
	SMBQ exhaustion	Burnout	Continuous		0-7
	SMBQ listlessness	Burnout	Continuous		0-7
	ISI	Insomnia severity	Interval		0-28
	PSS-10	Perceived stress	Interval		0-40
**Treatment-related predictors**					
	Clinician treatment expectancy	—	Interval	✓	0-10
	Treatment credibility scale	—	Interval		0-10
**Cognitive functioning**					
	SDMT^p^	Attention and processing speed	Interval		0-∞
	FAS^q^	Executive functions	Interval		0-∞
	Stroop index	Executive functions	Continuous		0-∞
	Stroop inhibition	Executive functions	Continuous		0-∞
	CERAD^r^ learning	Memory	Interval		0-30
	CERAD recognition	Memory	Interval		0-10
	Corsi forward	Memory	Interval		0-9

^a^Not applicable.

^b^S-ED: self-rated exhaustion disorder.

^c^ADNM-8: The Adjustment Disorder New Module-8.

^d^AUDIT: Alcohol Use Disorder Identification Test.

^e^GAD-7: General Anxiety Disorder-7.

^f^SMBQ: Shirom-Melamed Burnout Questionnaire.

^g^MADRS-S: Montgomery-Åsberg Depression Rating Scale.

^h^KEDS: Karolinska Exhaustion Disorder Scale.

^i^WHODAS: World Health Organization Disability Assessment Schedule.

^j^BBQ: Brunnsviken Brief Quality of Life Scale.

^k^ISI: Insomnia Severity Index.

^l^SRH-5: Self-Rated Health-5.

^m^PSS-10: Perceived Stress Scale-10.

^n^PHQ-15: Patient Health Questionnaire-15.

^o^6-QEMP: 6-item Questionnaire of Everyday Memory Problems.

^p^SDMT: Symbol Digit Modality Test.

^q^FAS: Verbal Fluency Test.

^r^CERAD: Consortium to Establish a Registry for Alzheimer’s Disease.

#### Sociodemographic Variables

Information on age (interval), sex (male, female, other, or prefer not to disclose), relationship status (in relationship, single, or widowed), number of children, educational attainment (in 9 categories between <9 years of school to PhD), employment status (eg, student, unemployed, or full-time work), and employment type (in 11 categories, eg, employed in the private sector, by the municipality, or other) was gathered before the start of treatment using the web-based study platform. Self-rated reading and computer skills were rated separately on a 5-step ordinal scale from *poor* to *very good*. Patients also reported if they were Swedish native speakers.

#### Clinical Characteristics

During the clinical interview, patients reported their medication regimen, specifying both the number (0-4) and type of medication (antidepressants, anxiolytics, sleep medication, and pain medication and yes or no). Primary diagnosis (AD or ED), and possible secondary psychiatric diagnosis (eg, anxiety or depressive disorder) was assessed by the clinician using Mini International Neuropsychiatric Interview, self-rated ED (ordinal categories ranging from *no* to *yes—to a high degree*) [32], and the ADNM-8 [33]. Using ADNM-8, the patient was asked about which specific stressors had been present in the past 2 years (in 16 options, eg, *too much or too little work* or *financial difficulties*). The clinician assessed the length of the current episode (in months), and the age of the patients first episode (in years). Sick-leave status upon inclusion in the study (0%-100% in 5 steps), length of current sick-leave episode (*0-1 months* to *>12 months* in 5 categories) was self-reported.

#### Self-Rated Symptoms

*Alcohol use* was assessed using the Alcohol Use Disorder Identification Test [40,41]. This 10-item screening instrument evaluates alcohol consumption, drinking behavior, and alcohol-related problems over the past year. It contains items, such as *How often do you have six or more drinks on one occasion*? rated on various ordinal scales, typically ranging from 0 to 4.

*Symptoms of anxiety* were measured using the Generalized Anxiety Disorder-7 scale [42]. This screening tool assesses generalized anxiety symptoms over the past 2 weeks. It comprises 7 items, such as *not being able to stop or control worrying* rated on a 4-point ordinal scale ranging from 0 *not at all* to 3 *nearly every day*.

*Symptoms of burnout* were measured using the Shirom-Melamed Burnout Questionnaire [43,44]. It aims to measure 3 components of burnout; emotional and physical fatigue, cognitive weariness, and listlessness and contains statements such as *I have difficulty concentrating* rated on a 7-point scale ranging from 1 *never or almost never* to 7 *always or almost always* with some items using reversed scoring.

*Symptoms of depression* were measured using Montgomery-Åsberg Depression Rating Scale [45]. It is a 9-item questionnaire used to measure different aspects of depression such as concentration difficulties, suicidal thoughts, sadness, and affected appetite with answers rated on a 7-point ordinal scale from 0 to 6.

*Symptoms of exhaustion disorder* were measured using the 9-item Karolinska Exhaustion Disorder Scale [46]. Measuring different aspects of exhaustion such as fatigue, endurance, and sleep impairment, answers are rated on an ordinal scale from 0 to 6 (eg, ability to concentrate; ranging from 0 “I do not have any difficulty concentrating, and can read, watch TV and converse normally” to 6 “I cannot concentrate on anything at all*.*”)

*Functional disability* was measured using The World Health Organization Disability Assessment Schedule (2.0) [47], developed to assess functioning in the last 30 days in 6 different life domains, including cognition, mobility, self-care, relationships, life activities, and societal participation. It contains statements, such as “I have difficulty standing for longer periods such as 30 minutes.” Answers are rated on a 5-point ordinal scale ranging from 0 *never* to 4 *extreme or unable*. A 12-item version was used.

*Quality of life* was assessed using the EQ-5D-5L [48,49] and the Brunnsviken Brief Quality of Life Scale [50]. The EQ-5D-5L contains 5 dimensions: mobility, self-care, usual activities, pain or discomfort, and anxiety or depression each rated on 5 levels of severity from *no problems* to *extreme problems*. The Brunnsviken Brief Quality of Life Scale is a 12-item questionnaire that assesses 6 life areas (leisure time, view on life, learning, creativity, view of self, and friends and friendship). Ratings range from 0 *strongly disagree*, to 4 *strongly agree*, on statements of the importance and satisfaction of each area.

*Insomnia severity* was measured using the Insomnia Severity Index [51]. The Insomnia Severity Index is a 7-item questionnaire designed to assess aspects of insomnia, including difficulty falling asleep, difficulty staying asleep, and satisfaction with sleep. Ratings are given using an ordinal scale ranging from 0 to 4.

*Self-rated health* was assessed using Self-Rated Health 5 asking patients to rate their general health on a scale of 1, *very bad* to 5 *very good* [52].

*Somatoform symptoms* were assessed using the Patient Health Questionnaire [53]*.* It consists of 15 questions covering somatic symptoms commonly seen in primary care, such as back pain, headache, and nausea. Answers are rated on a 3-point ordinal scale ranging from *not at all bothered* to *bothered a lot.*

*Subjective memory impairment* was measured using the 6-item Questionnaire of Everyday Memory Problems (6-QEMP) [54]. A 5-item version has previously been used to assess subjective memory problems in this patient population [55,56]. The present version was adapted by Stigsdotter Neely for use in patients with stress-related disorders with statements, such as “How do you think your memory functions now compared to before your stress-related mental health problems?.” The answers are rated on a 5-point ordinal scale.

#### Treatment-Related Predictors

*Clinician Treatment Expectancy* was judged after patient assessment, upon inclusion in the study, by clinicians rating the probability of the patient improving after treatment on a scale of 0 *no improvement* to 10 *full remission*.

The *Treatment Credibility Scale* was administered 3 weeks after the start of treatment [57]. Patients were asked questions about their impression of the treatment and if they thought they would improve. It included statements such as “How logical do you think this treatment is?” and “How confidently would you recommend this treatment to a friend with the same problems as you?” on a scale of 0 *not at all* to 10 *very logical*, *very confidently.*

#### Cognitive Functioning

*Attention and processing speed* were measured using the Symbol Digit Modality Test. A test originally developed by Smith [58,59] that measures visual detection, attention, and processing speed. A key with 9 different symbols and matching numbers is shown on the upper part of the display. At the center one of these 9 symbols are shown and the task of the participant is to choose the corresponding number using the key as guidance. The test score is the number of correct entries in 90 seconds. Comparable substitution tasks are considered sensitive to treatment effects for patients with multiple sclerosis [60] and depression [61], and it has been used in patients with stress-related disorders [62].

*Executive functioning* was measured using the Verbal Fluency Test (FAS) Word Fluency Test and the Stroop test. FAS was first described by Spreen and Benton [63], and it measures spontaneous verbal fluency and selective attention and shifting. The participant is tasked with producing words beginning with a certain alphabet letter (F, A, and S). Names, numbers, or repeated words are not allowed. The test score is the number of correct words beginning with the letter. FAS and similar word fluency tasks have been shown to be impaired in patients with stress-related exhaustion [62].

The Stroop test, originally developed by Stroop [64] and described by Jensen and Rohwer [65], measures executive functioning, inhibition, as well as updating and processing speed [66]. The test has 2 parts, (1) 20 color words are presented (green, yellow, blue, or red) and they are colored congruent to their meaning (eg, the word red colored in red). In the bottom part of the display, the color words are displayed on 4 buttons. The task is to, as quickly and thoroughly as possible, click the correct button. (2) Twenty color words are presented but displayed in an incongruent color (eg, the word red colored in green). The task of the participant is to click the button containing the color of the word as quickly and thoroughly as possible. Test score is calculated as an index (number of correct answers in part 2 divided by average time in seconds from part 2) and for interference (average time in part one–average time in part one). Performance of Stroop in patients with stress-related disorders has been shown to be impaired in 2 studies [62,67], but not in others [68,69].

*Memory and learning* were assessed using the Consortium to Establish a Registry for Alzheimer’s Disease (CERAD) Word List Learning Test and Corsi block-tapping test forward. CERAD was originally developed for use with Alzheimer disease [70] but is similar to other word-list tasks used in this patient population. It measures verbal learning and episodic memory. In the learning part of the test, a word list containing 10 words is presented over 3 trials and the task after every trial is to recall the words from the list. For every presentation the order is mixed. In the delayed recall part of the test (trial 4) that occurs after 5 to 10 minutes, the participant is asked to recall the words. Test score for the learning time is number of correct words in trial 1 to 3, and in the delayed recall part, number of correct words in trial 4. Similar word-list tasks have been used previously to assess memory functioning in patients with stress-related disorders [62,69].

Corsi block-tapping test forward gives information about visual ability of attention, short-term memory and working memory [71]. It contains 2 parts, but in this test battery, only the first part of the test is used. Nine blocks are displayed, and the testing platform starts by lighting up a sequence of blocks. The task is to repeat the sequence of blocks that the platform has displayed. The task starts out easy with only 2 blocks, but the difficulty increases by adding a longer sequence of blocks until the participant enters the incorrect sequence twice at the same number of blocks. The test score is the maximum number of correct repeated blocks. A cross-sectional study comparing patients with stress-related disorders to a healthy normative group found impaired performance on this test [62].

### Planned Statistical Analysis

All data will be prepared and analyzed using the latest version of Python [72] and the libraries NumPy [73], Pandas [74], and scikit-learn [75] or equivalent statistical packages. A notebook containing the analysis in documented code will be made available on Open Science Framework [76] for research transparency following the analysis.

#### Data Cleaning and Preparation

We will transform categorical variables into a format suitable for numerical analysis. For binary categorical variables, we will use label encoding. For multinomial variables, we will apply one-hot encoding. In addition, for ordinal data, which have a natural order, we will transform the categories into integers.

Predictor variables with over 20% missing data will be excluded from the analysis. Categorical variables exhibiting low variance, as determined by predictors with <5% of a certain response will be removed. For instance, by removing the variable “Sleep medication” if it only occurs in 3 out of 300 patients. This approach aims to reduce unnecessary complexity in the predictions and to minimize the risk of overfitting. To control for multicollinearity, variables with a correlation coefficient ≥0.8 will be removed from the traditional prediction analysis but will be retained for the ML model development. Data that are highly skewed will be transformed if deemed appropriate.

Cognitive test results will be manually reviewed before model fitting to validate a proper result. Comments pertaining to technical difficulties and disturbances that may have affected the test result will be assessed by two of the authors and lead to exclusion if so judged. Participants who have noted during screening that Swedish is not their native language will be excluded from the analysis for CERAD and FAS. We will standardize the raw scores from the cognitive tests using normative regression models with age, education and sex as covariates. This standardization process will convert raw scores into *Z* scores, as previously described by Franke Foyen et al [62] and for a full overview of the multiple linear regression models used and how they were calculated, see the studies by Mindmore [77] and van den Hurk et al [78].

Patients who have missing data for the posttreatment PSS-10, ie, the missing outcome variable for the primary aim, will be replaced by a PSS-10 process measurement at week 10 if available; If not, the patient will be excluded from the analysis. The number of participants excluded from the final models will be described.

To prepare our primary outcome, RCI for the PSS-10 before to after treatment will be computed using the following formula [39]:




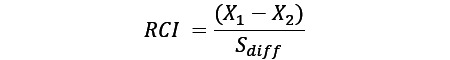







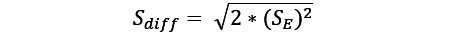







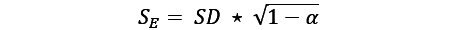




Cronbach α=0.83 from normative data will be used [38]. Patients exhibiting an RCI of −1.96 will be classified as responders.

#### Descriptive Statistics

Descriptive statistics will be used to summarize the sample characteristics and pretreatment variables, including mean or median, SDs and IQR for continuous variables, and proportions for categorical variables.

#### Predictor Analysis

For the traditional regression analysis, data will be imputed using the KNN imputer. The imputer, a nonparametric imputation method, works by imputing missing values based on the k-nearest neighbors; in this study *k* will be determined by cross-validation. It uses the Euclidean distance metric to find the nearest neighbors and can be used for both numerical and categorical data. Each missing value is imputed using values from its k-nearest neighbors. After imputation, we will run univariate logistic regressions for each predictor listed in Table 1 using RCI as a target variable. Predictors that are statistically significant in the univariate analyses will then be included in an ablation study, a systematic approach to evaluate feature importance. This method involves iteratively removing each significant predictor from a full model, measuring the change in explained variance, and then reinserting it, thereby quantifying each predictor’s unique contribution to the model’s explanatory power in the context of all other features.

#### ML Model Development

For an introduction on the technical terms introduced in this section, see the review article by Bzdok and Meyer-Lindenberg [13].

#### Train Test Split

As the ultimate goal of any model is to predict an outcome in unseen data, the ML models will be developed using a training set, and then evaluated on a test set stratified on main diagnosis (AD or ED) and responder status. In total, 70% of the data will be used for selecting predictor variables and training the models, and 30% for testing the prediction accuracy of the models. The choice of 70 to 30 was due to the limited size of our sample, as fewer observations in the testing data makes it difficult to use uncommon predictors. No external validation set is currently available at the time of writing.

#### Standardization and Imputation

Standardization and imputation will be applied on the training and test data separately to avoid data leakage. Numerical data will be standardized and all missing data will be imputed using the KNN imputer.

#### Model Descriptions

We will train and evaluate 4 different ML classifiers, a multiple logistic regression (LogReg) classifier using elastic net, a random forest (RF) classifier, a support vector machine (SVM) classifier, and an AdaBoost classifier. For a review of the models used, see the textbook by Geron [79]. In short, the LogReg classifier works by modeling the probability of a binary outcome based on one or more predictor variables, using the logistic function to ensure the output is between 0 and 1. We will use elastic net regularization to facilitate feature selection and prevent overfitting. Elastic net combines L1 (lasso) and L2 (ridge) penalties, encouraging sparsity and maintaining stability in the model. The RF classifier works by building multiple decision trees on random subsets of data and predictors. Each tree’s prediction is based on splits that minimize variance in the target variable, with the final model ensembling these predictions. The SVM classifier works by finding the hyperplane that maximizes the margin between different classes in the feature space. SVM is particularly effective in high-dimensional spaces and when the number of dimensions exceeds the number of samples. AdaBoost, the final classifier, works by combining multiple weak classifiers, typically decision trees, into a single strong classifier. It sequentially fits these weak learners on repeatedly modified versions of the data, focusing more on misclassified instances to improve overall accuracy.

#### Hyperparameter Tuning

We will conduct 5-fold cross-validation using randomized search for hyperparameter tuning and training evaluation to enhance the external generalizability and robustness of the results. This process involves defining a hyperparameter space, then randomly selecting a predetermined number of samples—in this case, 10—from this space, and conducting 5-fold cross-validation for each selected set of hyperparameters. Fivefold cross-validation is done by partitioning the data into 5 subsets, training the model on 4 subsets, and validating it on the remaining subset. This process is repeated 5 times, with each subset used exactly once as the validation data. The best performing hyperparameters will be chosen for the final models that are trained and then evaluated on the test set.

The hyperparameter ranges for the LogReg will include C values from 0.01 to 100 and l1_ratio values from 0 to 1. For RF, the parameter ranges will include the number of estimators from 5 to 1200, minimum samples required to split a node from 10 to 200, maximum depths from 5 to 750, and a binary indicator for bootstrapping. For SVM, the parameter range for the randomized search will include regularization parameter C values from 0.01 to 1 and for AdaBoost, the parameter ranges for the randomized search will include the number of estimators, ranging from 1 to 1500, and learning rates from 0.001 to 2.5.

#### Model Interpretation

The models developed to identify the responder status will be evaluated using BACC, precision and recall, both in the training set obtained through k-fold cross-validation and in the test set. Predictor importance in the RF model will be determined using Scikit-learn’s Feature importance function, which quantifies each predictor’s contribution to the model’s balanced classification accuracy. Area under the curve will be used to assess the models’ capability to distinguish between classes accurately. The approach will aim to provide a clear understanding of the models’ effectiveness and the role of various predictors. Our primary outcome of interest for comparison will be BACC in each model in the test set with the aim that (1) the model should perform better than a dummy model that simply predicts the most common responder status, and (2) that the model should perform 67% BACC or above to be deemed clinically useful [28]. Furthermore, the models will be statistically compared using bootstrap sampling. Specifically, we will generate 5000 bootstrap samples from the test set, calculating the BACC for each model on each sample. The distributions of these bootstrap BACCs will be compared and we will conclude that there is a statistically significant difference between models if the CIs do not overlap.

## Results

**Figure 1:**
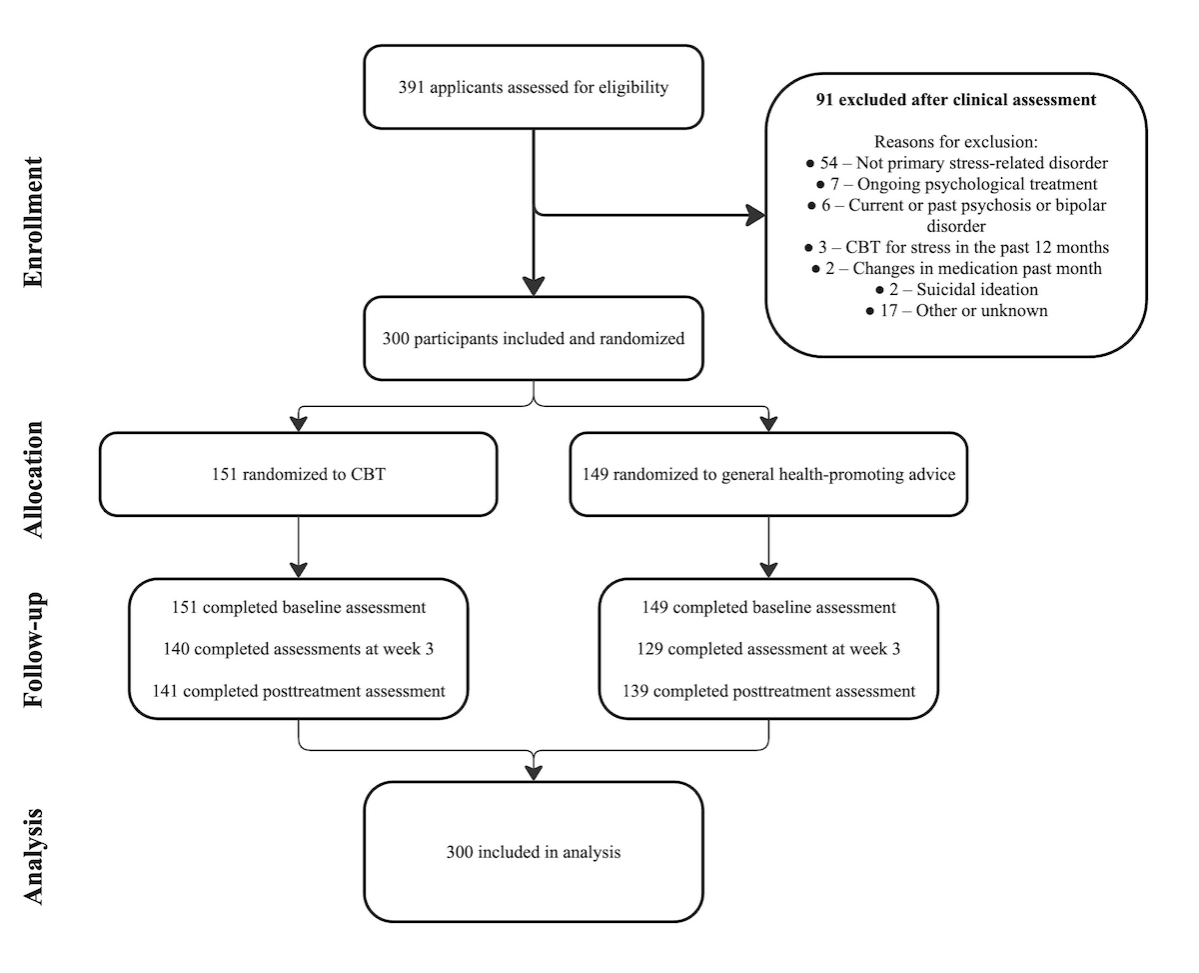
CONSORT (Consolidated Standards of Reporting Trials) diagram showing participant flow through enrollment, allocation, follow-up, and analysis. CBT: cognitive behavioral therapy.

This study was funded by ALF medicin (20190148), Region Stockholm (SLSO 2022–1278; SLSO 2022–1276), and Region Stockholm in collaboration with Stockholm university (FoUI‑939533). OF is supported by the Swedish innovation agency (No. 2022-00549). All data were collected (N=300) between April 2021 and September 2022. For a participant flow diagram throughout the study, see Figure 1.

A cross-sectional study investigating baseline cognitive functioning as compared with a healthy reference group has been published indicating small-to-moderate objective cognitive impairments [62], raising the question of whether objective cognitive function serves as a predictor of treatment response. In addition, an interim analysis of pre- and postcomparisons was presented at a conference in September 2022. These earlier analyses addressed separate research questions and did not influence the design, methods, or objectives of the current protocol. As of March 2025, data have not been analyzed for this study.

## Discussion

### Overview

This study will use a high-quality dataset from an RCT to investigate potential treatment predictors using both traditional prediction methods and an ML paradigm. This dual approach will enable the identification of predictors of treatment response in a patient population where prior research is limited. In addition, it will facilitate comparisons between different methodological approaches to prediction research.

### Comparison to Prior Work

To the best of our knowledge, this is the first study to apply an ML approach to study predictors of treatment outcome in patients diagnosed with AD or ED. In line with previous traditional prediction research of treatment outcomes in stress-related disorders, we hypothesize that younger age, education level, symptom severity, treatment credibility, and history of sickness absence will predict treatment response [15,22]. Furthermore, we anticipate that the ML models will outperform a dummy model and achieve a BACC of 67% or higher, surpassing the benchmark indicated by Forsell and others [28]. If confirmed, our findings would support the notion that predictive models using sociodemographic, clinical, self-rated, treatment-related, and potentially cognitive variables are valuable when predicting therapy outcomes, as have been suggested in other patient populations [17,18,80]. In subsequent research, these models should be externally validated and tested in implementation trials to assess their utility as decision support tools. Such trials could evaluate whether integrating predictions into treatment planning improves outcomes and supports personalized care.

### Strengths and Limitations

The study’s strengths include the use of a multimodal dataset from an RCT, including objective cognitive functioning. By comparing traditional prediction methods with advanced ML models and employing techniques, such as cross-validation and hyperparameter tuning, the study has the potential to generate robust and generalizable insights into treatment outcomes, contributing to methodological advancements in prediction research.

A limitation is the modest sample size (N*=*300) which may increase the risk of overfitting. While smaller sample sizes have been used in previous ML studies [80-82], it is well established that limited sample sizes can hinder generalization [23]. The minimal sample size required for ML prediction in mental health research depends on the explanatory power of the predictors, with some researchers advocating for at least 300 observations [83], while others recommend a larger sample of 500 to 1500 for studies involving predictors with low explanatory power [84]. External validation is widely regarded as the gold standard to ensure model generalizability [85], but such data are not currently available for this study. However, ongoing data collection by the research group may enable external validation in the near future. In the meantime, k-fold cross-validation on the training set and validation on a separate test set will be used to estimate and mitigate overfitting, providing a basis for model evaluation within the study’s constraints.

In addition, the recruitment strategy, which relied on social media, newspaper advertisements, and health care clinic referrals, may introduce selection bias and limit the generalizability of the findings. Participants recruited through these channels may not fully represent the broader population of individuals with stress-related disorders, potentially overrepresenting individuals with higher internet access, health literacy, or willingness to participate in internet-delivered interventions. These factors should be considered when interpreting the applicability of the study’s results to other settings or populations.

Finally, ML models, such as RF, while effective at handling complex datasets, often prioritize predictive performance at the expense of interpretability. Unlike traditional statistical methods, their inclusion of numerous variables can make it challenging to understand the relationships between predictors and outcomes, limiting their integration into clinical practice where transparency is essential. Efforts to address this, such as using feature importance metrics, will be necessary to bridge this gap moving forward.

### Implications for Clinical Practice

The study’s findings could significantly impact clinical practice by contributing to the limited research on predictors of treatment outcome for stress-related disorders. Given the current lack of a gold standard treatment for AD and ED, this research is particularly timely and relevant. The investigation into ML models for treatment outcome prediction may encourage future larger-scale studies and, potentially, the implementation of these models in clinical settings as decision support tools. These could help clinicians tailor treatments by integrating complex data, such as patient demographics, symptom severity, and treatment history, to recommend evidence-based options, guiding therapy selection, and monitoring progress in real time. By operationalizing predictive insights, decision support tools could enhance clinical precision, reduce trial-and-error in treatment, and improve patient outcomes for individuals with stress-related disorders.

## Abbreviations

AD: adjustment disorder
ADNM-8: Adjustment Disorder New Module-8
BACC: balanced accuracy
CBT: cognitive behavioral therapy
CERAD: Consortium to Establish a Registry for Alzheimer’s Disease
ED: exhaustion disorder
FAS: Verbal Fluency Test
ML: machine learning
PSS-10: Perceived Stress Scale-10
RCI: reliable change index
RCT: randomized controlled trial
RF: random forest
SVM: support vector machine
TRIPOD+AI: Transparent Reporting of a Multivariable Prediction Model for Individual Prognosis or Diagnosis+Artificial Intelligence

## Data Availability

The data referenced in this study protocol are not yet available, as the study has not yet been conducted. Once the study is completed, data may be made available on reasonable request, subject to compliance with Swedish law (the Swedish Ethical Review Act: 2003:460). For such requests, please contact the corresponding author.
